# Integrated Single Cell and Bulk RNA-Seq Analysis Revealed Immunomodulatory Effects of Ulinastatin in Sepsis: A Multicenter Cohort Study

**DOI:** 10.3389/fimmu.2022.882774

**Published:** 2022-05-11

**Authors:** Lin Chen, Senjun Jin, Min Yang, Chunmei Gui, Yingpu Yuan, Guangtao Dong, Weizhong Zeng, Jing Zeng, Guoxin Hu, Lujun Qiao, Jinhua Wang, Yonglin Xi, Jian Sun, Nan Wang, Minmin Wang, Lifeng Xing, Yi Yang, Yan Teng, Junxia Hou, Qiaojie Bi, Huabo Cai, Gensheng Zhang, Yucai Hong, Zhongheng Zhang

**Affiliations:** ^1^ Department of Critical Care Medicine, Affiliated Jinhua Hospital, Zhejiang University School of Medicine, Jinhua, China; ^2^ Department of Emergency, Zhejiang Provincial People’s Hospital, People’s Hospital of Hangzhou Medical College, Hangzhou, China; ^3^ The 2nd Department of Intensive Care Unit, The Second Affiliated Hospital of Anhui Medical University, Hefei, China; ^4^ Department of Critical Care Medicine, The First People’s Hospital of Changde City, Changde, China; ^5^ Department of Emergency Medicine, The First Affiliated Hospital of Harbin Medical University, Harbin, China; ^6^ Department of Critical Care Medicine, Zhuzhou Central Hospital, Zhuzhou, China; ^7^ Emergency Department, Shengli Oilfield Central Hospital, Dongying, China; ^8^ Department of Critical Care Medicine, The Second Affiliated Hospital of Xi’an Medical University, Xi’an, China; ^9^ Department of Critical Care Medicine, Lishui Center Hospital, Lishui, China; ^10^ Department of Critical Care Medicine, The Fourth Affiliated Hospital of Anhui Medical University, Anhui Medical University, Hefei, China; ^11^ Department of Emergency Medicine, Sir Run Run Shaw Hospital, Zhejiang University School of Medicine, Hangzhou, China; ^12^ Department of Emergency Medicine, The Second Hospital of Jiaxing, Jiaxing, China; ^13^ Department of Critical Care Medicine, The First Affiliated Hospital of Xi’an Jiaotong University, Xi’an, China; ^14^ Department of Emergency, Qingdao Municipal Hospital, QingDao University School of Medicine, Qingdao, China; ^15^ Department of Critical Care Medicine, Second Affiliated Hospital, Zhejiang University School of Medicine, Hangzhou, China; ^16^ Key Laboratory of Precision Medicine in Diagnosis and Monitoring Research of Zhejiang Province, Department of Emergency Medicine, Sir Run Run Shaw Hospital, Zhejiang University School of Medicine, Hangzhou, China

**Keywords:** sepsis, single cell, ulinastatin, RNA-seq, immunosuppression

## Abstract

Sepsis is a leading cause of morbidity and mortality in the intensive care unit, which is caused by unregulated inflammatory response leading to organ injuries. Ulinastatin (UTI), an immunomodulatory agent, is widely used in clinical practice and is associated with improved outcomes in sepsis. But its underlying mechanisms are largely unknown. Our study integrated bulk and single cell RNA-seq data to systematically explore the potential mechanisms of the effects of UTI in sepsis. After adjusting for potential confounders in the negative binomial regression model, there were more genes being downregulated than being upregulated in the UTI group. These down-regulated genes were enriched in the neutrophil involved immunity such as neutrophil activation and degranulation, indicating the immunomodulatory effects of UTI is mediated *via* regulation of neutrophil activity. By deconvoluting the bulk RNA-seq samples to obtain fractions of cell types, the Myeloid-derived suppressor cells (MDSC) were significantly expanded in the UTI treated samples. Further cell-cell communication analysis revealed some signaling pathways such as ANEEXIN, GRN and RESISTIN that might be involved in the immunomodulatory effects of UTI. The study provides a comprehensive reference map of transcriptional states of sepsis treated with UTI, as well as a general framework for studying UTI-related mechanisms.

## Introduction

Sepsis is a leading cause of morbidity and mortality in the intensive care unit (ICU), with an estimated mortality rate of 10 – 50% depending on the severity of the illness (1, 2). Although sepsis is initially caused by infection, subsequent organ dysfunction is the result of uncontrolled inflammatory response. Thus, strenuous effects have been made to develop immunomodulatory drugs, such as corticosteroids, topoisomerase inhibitors, rhodomeroterpene and pterostilbene (3–7). However, few agents have been proven to be clinically effective in reducing sepsis outcomes. The sepsis comprises a heterogenous population, and different subtypes of sepsis may respond differently for a particular treatment (8, 9). Therefore, it is of vital importance to understand the biological mechanisms underlying the pathogenesis of sepsis from the perspective of system biology.

Ulinastatin (UTI) is a broad-spectrum serine protease inhibitor with potent immunomodulatory effects. Clinical evidence showed that UTI had a significant effect on inflammatory biomarkers such as C-reactive protein (CRP), interleukin-6 (IL-6), and tumor necrosis factor-alpha (TNF-α), as well as reduced and prevented MODS and lowered mortality in acute pancreatitis (10, 11). However, the underlying mechanisms of the immunomodulatory effects remain controversial, and a variety of biological pathways have been identified for being associated with UTI . However, none of these studies have explored these potential mechanisms from the perspective of system biology by exploiting a holistic approach to deciphering the complexity of biological systems that starts from the understanding that the networks that form the whole of living organisms are more than the sum of their parts. The high throughput second generation RNA sequencing (RNA-Seq) provides an unbiased approach to exploring the changes in transcriptome under different diseases status or treatment conditions (12). The current study explored potential underlying mechanisms of UTI on sepsis by integrating bulk and single cell RNA-seq data. The study provides a comprehensive reference map of transcriptional states of sepsis treated with UTI, as well as a general framework for studying UTI-related mechanisms.

## Methods

### Study Population and Setting

The study was conducted in 13 tertiary care hospitals in mainland China from Nov 2020 to August 2021. Patients fulfilling the sepsis-3.0 criteria (suspected or documented infection plus acute increase in SOFA score > 2 points) on admission to ED were potentially eligible for the present study (13). Subjects were excluded if they met one of the following criteria: 1) end-stage cirrhosis with Child-Pugh C; 2) pregnancy; 3) concomitant malignancy, autoimmune disease; 4) patients who signed do-not-resuscitate order; 5) sepsis onset > 48 hours or had been treated in other hospitals when presenting to the participating hospitals; 6) immunosuppression such as long-term use of immunosuppressive agents, chemotherapy, corticosteroids, radiotherapy or HIV infection; and 7) acute myocardial infarction and/or pulmonary embolism. The study was approved by the ethics committee of Sir Run Run Shaw hospital (approval number: 20201014-39).

Demographics and baseline characteristics including age on admission, sex, height, weight, comorbidities and site of infection were obtained on the day of admission. Other clinical and laboratory variables such as lactate, C-reactive protein (CRP) were obtained on day 1, 3 and 5. All patients were followed for mechanical ventilation, continuous renal replacement therapy (CRRT) and vasopressor dependent days during the hospital stay. The use of UTI (Techpool Bio-Pharma Co., Ltd.) and its dosage (300,000 to 1000,000 U/day) were determined by the treating physician. The standard dosage of UTI was 100,000 U once to three times per day. However, there is a few evidence that large dose UTI can have additional benefits (14, 15). Blood samples of 2 ml were drawn on day 1, 3 and 5 after hospital admission and processed within 6 hours. The raw data were deposited in the National Genomic Data Center (https://ngdc.cncb.ac.cn/bioproject/browse/PRJCA006118).

### Bulk RNA-Seq Library Preparation and Quantification

Peripheral blood mononuclear cells (PBMC) were isolated by using density-gradient centrifugation as per standard protocol. Total RNA was extracted and purified using TRIzol reagent (Invitrogen, Carlsbad, CA, USA) following the manufacturer’s procedure and were then stored at -80°. Then all samples were sent for library preparation and gene expression quantification (LC-BioTechnologies (Hangzhou) Co., LTD.). The remaining RNAs were reverse-transcribed to cDNA following ribosomal RNAs removal. U-labeled double-stranded DNAs was then synthesized with E. coli DNA polymerase I, RNase H and dUTP. The fragments were ligated with single-or dual-index adapters, and a size selection assay was performed with AMPureXP beads. U-labeled double-stranded DNAs were treated with heat-labile UDG enzyme. The ligated products were amplified with PCR under pre-established conditions. The average insert size for the final cDNA library was 300 bp ( ± 50 bp). Finally, we performed paired-end sequencing on an Illumina NovaSeq™ 6000 following the vendor’s recommended protocol.

The reads containing adapter contamination, low-quality bases and undetermined bases were removed using cutadapt-1.9 (cutadapt.readthedocs.io/en/stable/) (16). Then sequence quality was verified using FastQC v0.10.1 (www.bioinformatics.babraham.ac.uk/projects/fastqc/). We used HISAT2-2.0.4 to map reads to the genome of *Homo sapiens* obtained from the Ensembl v96 database (17). The mapped reads of each sample were assembled using StringTie-1.3.4 with default parameters (18). Then, the Gffcompare tool was used to compare and merge different gene annotations from all samples (19). After the final transcriptome was generated, the expression level of the RNAs were determined using StringTie to obtain FPKM values.

Differential gene expressions between the UTI and control group were tested by using negative binomial generalized linear models in the DESeq2 workflow (20). Clinical variables such as age, gender, SOFA, hospital days and interaction between days and UTI use were included in the model. The batch effects resulted from different hospitals were removed in the model. Weighted correlation network analysis (WGCNA) was used for finding modules of highly correlated genes, for summarizing such clusters using the module eigengene or an intramodular hub gene, for relating modules to one another and to external sample traits (using eigengene network methodology), and for calculating module membership measures (21). Methodological details for WGCNA can be found in electronic digital content. The correlation between module eigengene and UTI group was explored. Gene set enrichment analysis with gene ontology (GO) terms was performed for the modules highly correlated with the UTI group (22). All subontologies including cellular component, biological process, and molecular function were used for enrichment analysis. Regulatory mechanisms of the modules highly correlated with UTI were further explored with transcription factor (TF) enrichment analysis and microRNA correlation analysis (23, 24). Regulatory networks between TF/microRNA and candidate genes were matched using the *multiMiR* package (version 1.14.0).

### Single Cell RNA-Seq Quantifications and Statistical Analysis

Blood samples were processed for Singleron Matrix™ Single cell RNA sequencing. Briefly, Single-cell suspensions with 1×10^5^ cells/mL in concentration in PBS (HyClone) were prepared. Single-cell suspensions were then loaded onto microfluidic devices and scRNA-seq libraries were constructed according to Singleron GEXSCOPE^®^ protocol by GEXSCOPE^®^ Single-Cell RNA Library Kit (Singleron Biotechnologies). Individual libraries were diluted to 4nM and pooled for sequencing. Pools were sequenced on Illumina HiSeq X with 150 bp paired end reads.

Raw reads were processed to generate gene expression profiles using an internal pipeline. Briefly, after filtering read one without poly T tails, cell barcode and UMI was extracted. Adapters and poly A tails were trimmed (fastp V1) before aligning read two to GRCh38 with ensemble version 92 gene annotation (fastp v2.5.3a and featureCounts v1.6.2) (25). Reads with the same cell barcode, UMI and gene were grouped together to calculate the number of UMIs per gene per cell. The UMI count tables of each cellular barcode were used for further analysis. Cell type identification and clustering analysis using Seurat (v4.0.4) and clustermole (v1.1.0) (26, 27). Rigorous quality control was performed and poor quality cells with the number of gene features >2500 or < 200, percent of mitochondria genes > 20% were removed. Standard Seurat workflow analysis was performed. Different samples were integrated with the IntegrateData function (26). The parameter resolution was set to 0.15 for FindClusters function to identify clusters. Differentially expressed genes (DEGs) between different clusters were identified with function FindMarkers, and gene set enrichment analysis (GSEA) was performed to identify enriched pathways (28).

### Deconvolution of Bulk RNA-Seq Data

The bulk RNA-seq data were deconvoluted by using the CIBERSORT algorithm (29), so that the fraction of each cell component can be estimated for the bulk RNA-seq samples. CIBERSORT exploited nu–support vector regression (*v-SVR*) to identify cell types by estimating relative subsets of RNA transcripts. The differences in the fraction of cell types between UTI and control samples were compared and visualized with violin plots (30).

### Cell-Cell Communication Inference and Intercellular Communication Networks

We inferred cell-cell communication using the CellChat (http://www.cellchat.org/) workflow (31). Specifically, the probability of cell-cell communications was inferred by integrating gene expression profile of each cell and prior knowledge of interactions between receptors, ligands, and their cofactors. Label-based mode was employed with cells labeled by the cell types obtained from the above Seurat workflow. The curated signaling molecule interaction database CellChatDB was used to assign roles for the signaling molecules and their interactions. Significant cell communications were obtained by identifying differentially over-expressed ligands and receptors for each cell group. The global communication patterns and key signals in different cell groups were identified by a pattern recognition method based on non-negative matrix factorization.

## Results

### Study Population

A total number of 145 sepsis patients were enrolled during the study period, including 22 patients in the UTI group and 123 in the control group. The UTI groups showed higher SOFA score [median (Q1, Q3): 7 (6-13) vs. 7 (5 - 8); p = 0.033] and serum lactate [5.2 (3.0 – 7.3) vs. 2.5 (1.6 – 3.9); p = 0.001] than the control group at baseline. Although the UTI group showed significantly higher serum lactate than the control group on admission, they had the similar vasopressor dependent days (p = 0.422), supporting the clinical and laboratory observations that UTI could mitigate septic shock (32, 33). Other baseline variables such as site of infection, comorbidities and use of MV were comparable between the two groups (**Table 1**).

**Table 1:** Baseline characteristics of the UTI and control groups.

Variables	Total (n = 145)	Control (n = 123)	UTI (n = 22)	p
**Age in years, Median (Q1, Q3)**	72 (62, 81)	74 (63.5, 82)	67 (53, 72)	0.009
**Sex, Male (%)**	87 (60)	74 (60)	13 (59)	1
**Infection Site, n (%)**				0.457
** Abdomen**	39 (27)	35 (28)	4 (18)	
** Biliary/Liver**	16 (11)	13 (11)	3 (14)	
** Brain**	3 (2)	3 (2)	0 (0)	
** Intestine**	8 (6)	7 (6)	1 (5)	
** Lung/Chest**	33 (23)	30 (24)	3 (14)	
** Others**	6 (4)	4 (3)	2 (9)	
** Soft Tissue**	8 (6)	7 (6)	1 (5)	
** Urinary**	32 (22)	24 (20)	8 (36)	
**SOFA, Median (Q1, Q3)**	7 (5, 9)	7 (5, 8)	7 (6, 13)	0.033
**Height (cm), Median (Q1, Q3)**	165 (159.5, 172)	165 (160, 171)	164.5 (158.25, 174.25)	0.981
**Weight (kg), Median (Q1, Q3)**	62 (56, 70)	61 (57, 70)	65 (56.75, 70)	0.492
**Diabetes mellitus, n (%)**	33 (23)	30 (24)	3 (14)	0.405
**Hypertension, n (%)**	64 (44)	55 (45)	9 (41)	0.922
**Cardiac failure, n (%)**	14 (10)	12 (10)	2 (9)	1
**Use of MV, n (%)**	71 (49)	62 (50)	9 (41)	0.556
**Lactate (mmol/L), Median (Q1, Q3)**	2.7 (1.6, 4.53)	2.5 (1.6, 3.86)	5.18 (2.92, 7.28)	0.001
**CRP (mg/dl), Median (Q1, Q3)**	128.56 (60.87, 200.68)	122.88 (62.56, 198.5)	176.04 (59.16, 250.44)	0.171
**MV days, Median (Q1, Q3)**	2 (0, 7)	2 (0, 8)	1.5 (0, 5.75)	0.352
**CRRT days, Median (Q1, Q3)**	0 (0, 0)	0 (0, 0)	0 (0, 3.75)	0.021
**Vasopressor dependent days, Median (Q1, Q3)**	2 (0, 6)	2 (0, 5)	3.5 (0, 6)	0.422

Q1, the first quartile; Q3, the third quartile; MV, mechanical ventilation; CRP, C-reactive protein; UTI, ulinastatin; SOFA, sequential organ failure assessment; CRRT, continuous renal replacement therapy.

### Differential Gene Expression in Bulk RNA-Seq Samples

DESeq2 standard workflow was employed to explore the differential expressed genes between the UTI and control groups. Since there were baseline difference in the cohort, we adjusted for the SOFA, age, sex and days in the negative binomial regression models. There are greater number of genes being suppressed than the number of being activated in UTI versus control groups (**Figure 2A**). These suppressed genes were significantly enriched in the Fc receptor signaling pathways, phagocytosis, neutrophil degranulation, and neutrophil mediated immunity (**Figure 2B**). Many genes involved in neutrophil mediated immunity were established to play important roles in the pathogenesis of sepsis, such as MAPK14 (3, 34), TLR2 (35, 36), FPR2 (37) and ITGAM (38). Interestingly, pathways involving neutrophil activation and degranulation were clustered together to form the largest cluster of top enriched pathways (**Figure 2D**). Many genes showed significant interactions between days and UTI treatment, indicating differential effect of UTI across day 1, 3 and 5 (**Figure 2F**). For instance, SERPINI2 showed higher expression levels in UTI group versus control group on day 1, but with declining expression levels through day 3 to 5 (**Figure 2G**), indicating it may take several days for UTI to take the modulatory effect.

### Weighted Gene Co-Expression Network Analysis (WGCNA) of Sepsis Gene Expression Profile

**Figure 1:**
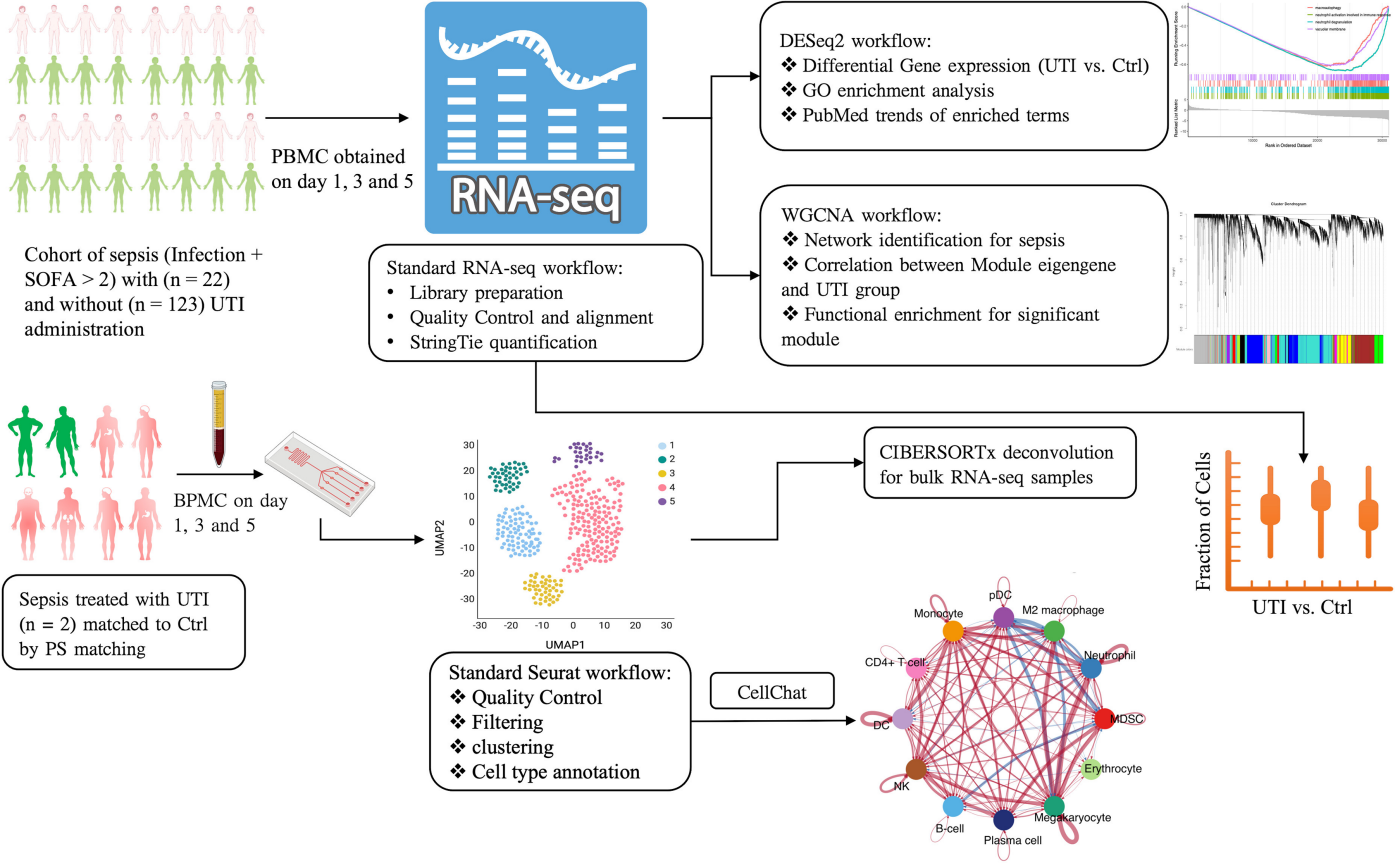
Schematic illustration of the integrated analysis of bulk and single cell RNA-seq analysis. PBMC, peripheral blood mononuclear cells; UTI, ulinastatin; SOFA, sequential organ failure assessment; GO, gene ontology; PS, propensity score; MDSC, Myeloid-derived suppressor cells; WGCNA, Weighted correlation network analysis; UMAP, Uniform manifold approximation and projection; pDC, Plasmacytoid dendritic cells; DC, dendritic cells; NK, natural killer.

**Figure 2:**
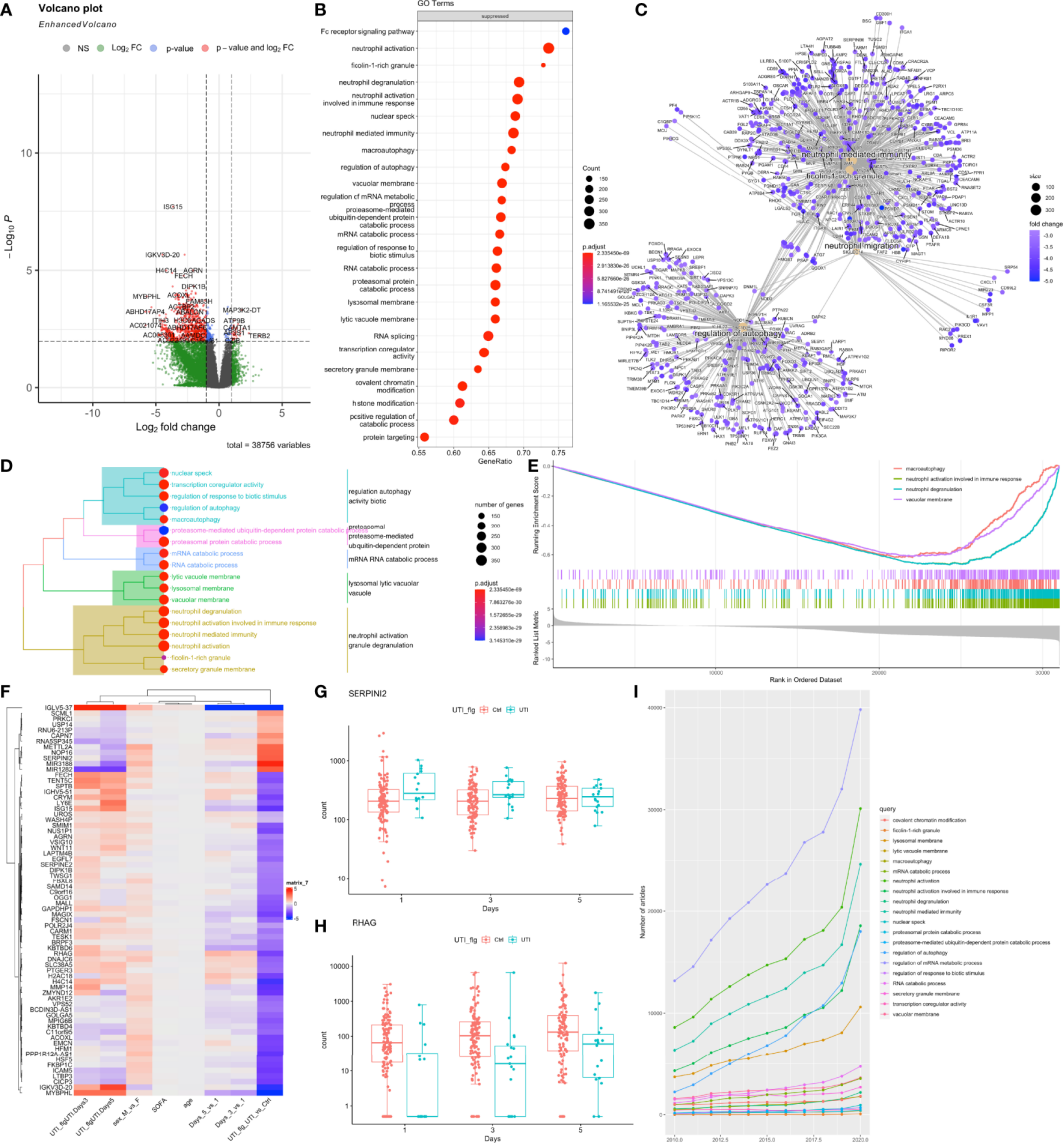
Differential gene expression analysis of bulk RNA-seq data. **(A)** Volcano plot showing the differentially expressed genes between UTI and control samples, while adjusting for SOFA, age, sex and days. P values were adjusted for multiple comparisons. Genes with adjusted p < 0.01 and log2 fold change > 1 were labelled. **(B)** gene set enrichment analysis of suppressed genes for GO terms. **(C)** network visualization of enriched terms and associated genes. **(D)** tree plot showing the hierarchical clustering of enriched terms by using Jaccard’s similarity index. **(E)** The GSEA enrichment score for several sample pathways. The running sum deviated in the negative direction suggesting suppressed pathways. **(F)** Heatmap showing the coefficient matrix of the negative binomial regression model, with an interaction term between days and UTI. Top 70 genes of the largest absolute values of coefficients were displayed. **(G, H)** Example genes with significant interaction effects between days and UTI groups are displayed with box plots. **(I)** publication trends of the top enriched terms extracted from PubMed search engine.

**Figure 3:**
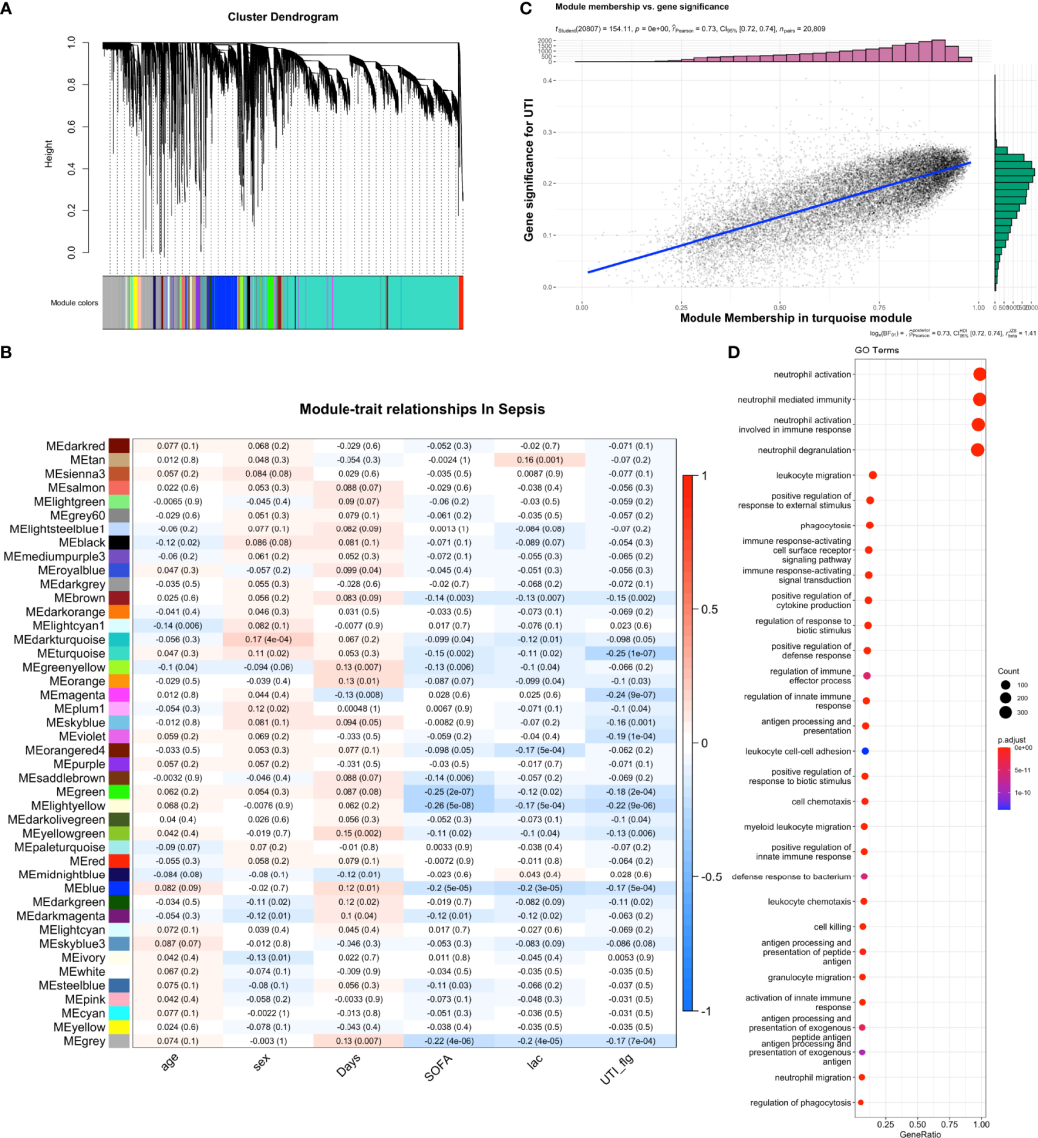
Weighted gene co-expression network analysis (WGCNA) of bulk RNA-seq samples. **(A)** Dendrogram clustering of the co-expressed genes into modules. Modules were labeled by different colors. **(B)** module-trait relationship in sepsis samples. The blue color indicates negative correlation and the red color indicates the positive correlation. The numbers in each cell are Pearson’s correlation coefficients between module eigengene and trait values, with p values shown in the parenthesis. **(C)** Correlation between module membership and gene significance for UTI. Each dot represents a gene. Gene significance was quantified by comparing gene expression between UTI and control groups. Module membership of a given gene was quantified by correlating its expression profile with the module eigengene. **(D)** Gene set enrichment analysis of the turquoise module with GO terms.

WGCNA identified multiple gene co-expression networks (**Figure 3A**; **Figures 1**, **2**). Most modules showed suppressed expression in the UTI group versus control group. In particular, the eigengene of the turquoise module was significantly correlated to the UTI group (correlation coefficient -0.25, p < 0.001, **Figure 3B**). The red module was also significantly suppressed by the UTI. The association between turquoise module and UTI group was confirmed by the correlation between module membership and gene significance for UTI (*R_pearson_* =0.73; p < 0.001, **Figure 3C**). GO term enrichment analysis showed that pathways including neutrophil activation, neutrophil degranulation and leukocyte migration were among the most enriched pathways for the turquoise module (**Figure 3D**).

### Regulatory Transcription Factor and miRNA Enrichment Analysis

**Figure 4:**
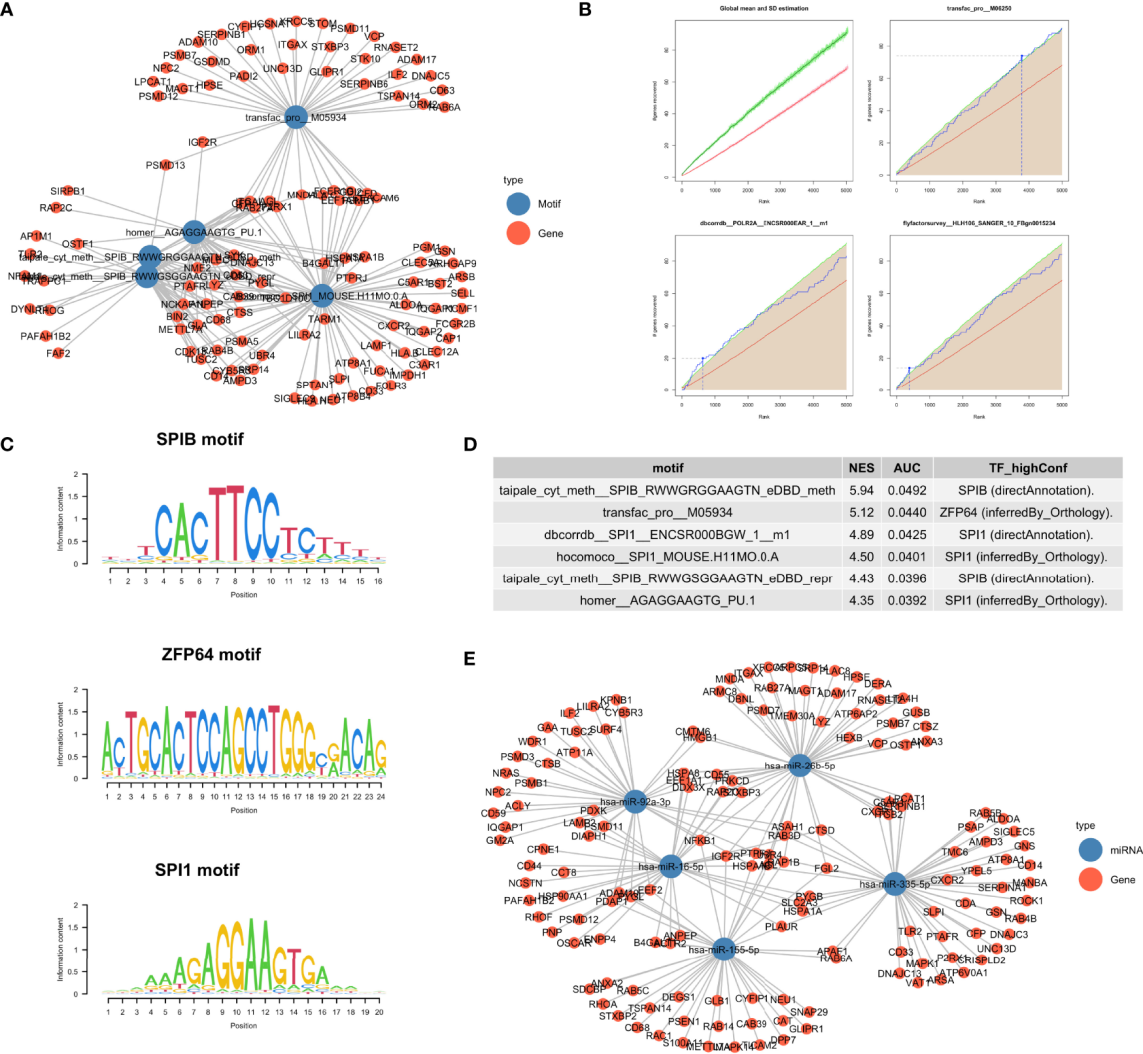
Regulatory drivers of the turquoise module. **(A)** Network visualization of the enriched motifs and genes. **(B)** Cumulative recovery curve for several example motifs. The red line represents the global mean of the number of recovered genes and the green line represents the third standard deviation (3SD). Motifs greater than the 3SD were considered statistically significant. **(C)** Sequence logo of enriched motifs, the relative width of a position is represented by the information content matrix. **(D)** Results of the top six enrichment motifs. **(E)** Network visualization of the top 5 miRNA and their target genes identified from the “mirtarbase” table.

To further explore how the enriched pathways were regulated by upstream molecular events, we explored motif enrichment for the turquoise module (**Figures 3**, **4**). The most significantly enriched motifs included aipale_cyt_meth:SPIB_RWWGRGGAAGTN_eDBD_meth (NES = 5.94), transfac_pro:M05934 (NES = 5.12) and dbcorrdb:SPI1:ENCSR000BGW_1:m1 (NES = 4.89). Transcription factors associated with these motifs included SPIB, ZFP64 and SPI1 (**Figure 4D**). Our results support previous finding that SPIB plays an important role in maintaining immune homeostasis (39). While the transcription factor Zinc Finger Protein 64 (ZFP64) has been explored in cancer metastasis (40), its role in sepsis has not been fully defined.

Several microRNA including hsa-miR-335-5p, hsa-miR-16-5p, hsa-miR-26b-5p and hsa-miR-155-5p were identified as the key regulators of the turquoise module involving neutrophil activation and degranulation (**Figure 4E**).

### Single Cell RNA-Seq Showing Expansion of MDSC in UTI Group

**Figure 5:**
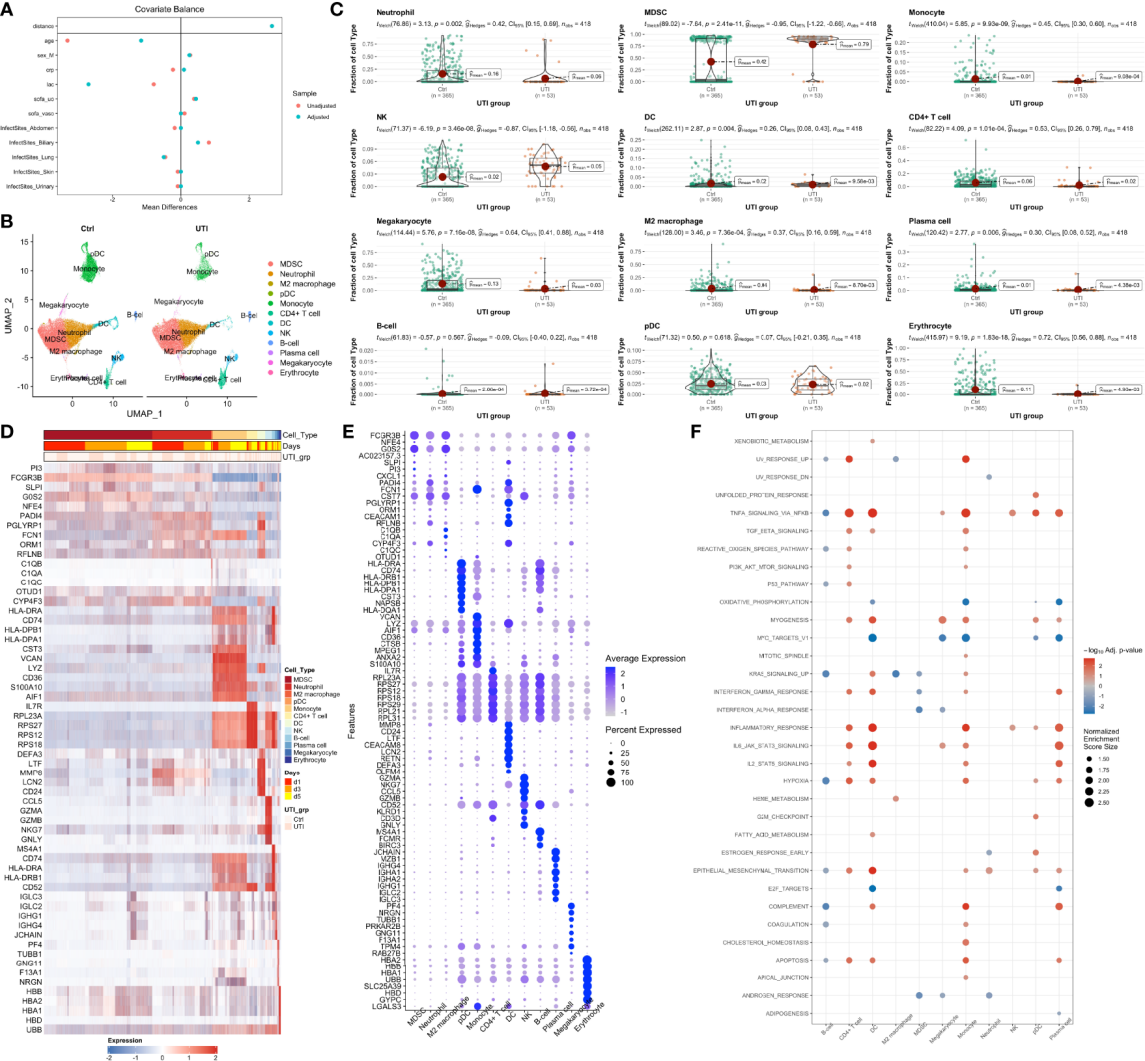
Single cell RNA-seq analysis in UTI and control samples. **(A)** balance diagnostics of propensity score matching for samples with and without UTI administration. **(B)** Uniform manifold approximation and projection (UMAP) of single cell gene expression profile, stratified by UTI groups. Twelve cell types were identified for the 103,870 cells. **(C)** Comparisons of the cell type fractions between UTI and control groups. Cell type fractions of the bulk RNA-seq samples were inferred by deconvolution methods. The effect size of ${\hat{g}}_{hedge}$ is calculated by dividing the mean difference with pooled standard deviation. **(D)** Heatmap and **(E)** dot plot showing the marker genes of each cell type. **(F)** Gene set enrichment analysis of the differentially expressed genes between UTI and control groups across all cell types.

**Figure 6:**
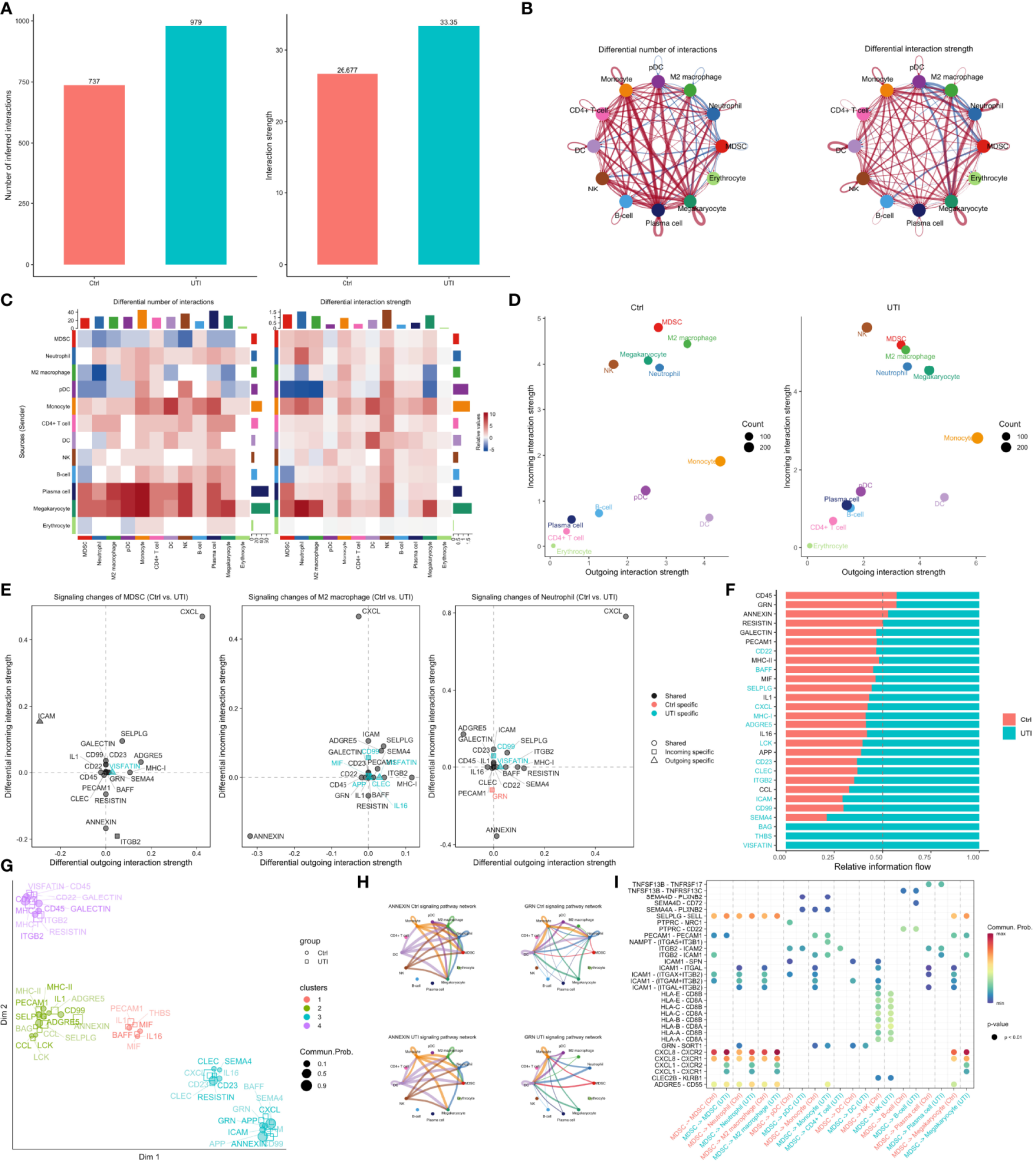
Comparisons of cell-cell communications between UTI and control groups. **(A)** The number and strength of inferred communication links between UTI and control groups. **(B)** Circle plot showing differential cell-cell communication networks between UTI and control groups. The width of edges represents the relative number of interactions or interaction strength. Red (or blue) colored edges represent increased (or decreased) signaling in the UTI compared to control. **(C)** Heatmap showing differential number of interactions or interaction strength in the cell-cell communication network between control and UTI groups; red color indicates increased signaling in the UTI compared to control. The top colored bar plot represents the sum of column of values displayed in the heatmap. The right colored bar plot represents the sum of row of values. **(D)** Scatter plots showing the dominant senders (sources) and receivers (targets) in a 2D space. x-axis and y-axis are respectively the total outgoing or incoming communication probability associated with each cell group. Dot size is proportional to the number of inferred links (both outgoing and incoming) associated with each cell group. Dot colors indicate different cell groups. **(E)** 2D visualization of differential outgoing and incoming signaling associated with one cell group. Positive values indicate the increase in the UTI dataset while negative values indicate the increase in the control dataset. **(F)** Ranking signaling networks based on the information flow. Significant signaling pathways were ranked based on differences in the overall information flow within the inferred networks between sepsis and septic shock. The top signaling pathways colored red are enriched in control, and these colored green were enriched in UTI. **(G)** 2D visualization of the joint manifold learning of signaling networks from UTI and control datasets. Each dot represents the communication network of one signaling pathway. Dot size is proportional to the overall communication probability. **(H)** Circle plot showing the inferred signaling network at UTI and control groups. Edge width represents the communication probability, and the edge colors are consistent with the color of the sender cell type. **(I)** Comparisons of significant interactions (Ligand-Receptor pairs) between UTI and control, which contribute to the signaling from MDSC to M2 macrophage, monocyte, megakaryocytes, and neutrophil subpopulations. Dot color reflects communication probabilities and dot size represents computed p-values. Empty space means the communication probability is zero. p-values are computed from one-sided permutation test.

Two patients received UTI treatment were matched to corresponding two control subjects by propensity score (**Figure 5A**). A total of 103,870 PBMCs were harvested and passed quality control (**Figures 4**, **5**), which were clustered into 12 cell types including Myeloid-derived suppressor cells (MDSC), neutrophil, M2 macrophage, Plasmacytoid dendritic cells (pDC), monocytes, CD4+ T cell, dendritic cells (DC), natural killer (NK) cell, B-cell, plasma cell, megakaryocyte, and erythrocyte (**Figure 5B** and **6**). To explore the changes of cell fractions with the treatment of UTI, the bulk RNA-Seq samples were deconvoluted into cellular components using the CIBERSORT method (41). Fifty-three PBMC samples from 22 patients treated with UTI were compared to 365 control samples. While neutrophil fraction was significantly reduced with UTI treatment $\left(\text{standardized mean difference }{\hat{g}}_{hedge}=0.42[0.15,0.69];\text{p}=0.002\right)$ , the MDSC fraction was significantly expanded in the UTI group $\left[{\hat{g}}_{hedge}=-0.95(-1.22,-0.66);\text{p}=0.002\right]$ (-1.22, -0.66); p = 0.002]. MDSCs constitute a heterogeneous population of immature myeloid cells that potently suppress immune responses, and is enriched in sepsis (42–44). However, the regulatory mechanism of MDSC expansion in sepsis is unknown. Our study provides evidence that UTI may take their immunomodulatory effects *via* regulating MDSC expansion. Transcriptomic biomarkers of each cell type were explored by comparing the differential gene expression for a specific cell type versus all other remaining cell types (**Figures 5D, E**). Consistent with that reported in the literature (45), transcriptomic biomarkers of MDSC included PI3 and SLPI. However, there are significant overlap of transcriptome profiles between neutrophils and MDSC, raising the question whether human MDSCs and neutrophils are actually different cell types or whether they are one plastic cell type that can functionally polarize from microbial killers to immunosuppressor cells (46).

By comparing gene expression profiles between UTI and control groups across all cell types, some significantly enriched pathways were identified (**Figure 5F**). For example, the interferon-gamma and alpha response pathways were suppressed in the MDSC by UTI treatment. Many pathways display cell specific activity. While TNF-alpha signaling *via* NF-*_κ_* B was upregulated in monocytes, DC and CD4+ T cells, it was down-regulated in B-cells, indicating the suppression of humoral immunity and activation of cellular immunity. Interestingly, our study showed that the estrogen response in neutrophil is suppressed, consistent with findings from the bulk RNA-seq analysis showing that many neutrophil mediated processes are suppressed in the UTI group. There has been evidence that suppression of estrogen receptor can inhibit the neutrophil extracellular traps formation (47).

### Difference of Cell-Cell Communications Between UTI and Control Samples

To further explore the difference of cell-cell communications between UTI and control samples, the CellChat method was employed to infer communications between cell types (31). Overall, there was greater number and strength of cell-cell communications in the UTI samples than the control samples (**Figure 6A**). However, the signals in neutrophil and MDSC were decreased in both the number and strength (**Figure 6B**). More specifically, the signals of neutrophils and MDSC as the receiver were decreased. However, signals from neutrophil (sender/source) were not significantly reduced (**Figure 6C**), supporting the notion that neutrophils are effector cells. In contrast, MDSC is acting as a modulatory cells that send more immunosuppressive signals to other cells in the UTI group than the control group. The specific signaling pathways were explored in more details in MDSC, neutrophil and M2 macrophage (**Figure 6E**). The signaling pathways such as ANNEXIN, ITGB2, and RESISTIN were found to be reduced in the UTI group. ANNEXIN may play a detrimental role in sepsis, by prolonging neutrophil survival, which is known to contribute to sepsis-mediated organ damage (48, 49). RESISTIN signaling was suppressed in both MDSC and M2 macrophage by the use of UTI, supporting the therapeutic effects of the UTI. Elevated RESISTIN signaling has been associated with adverse outcome in sepsis in the Albumin Italian Outcome Sepsis (ALBIOS) trial (50). The above mentioned signaling pathways were altered between UTI and control groups, which was also supported by the clustering of each signaling in a 2D space (**Figure 6G**).

## Discussions

The study integrated bulk and single cell RNA-seq data to systematically explore the potential mechanisms of the effects of UTI in sepsis. After adjusting for potential confounders, such as age, gender, SOFA, hospital days and interaction between days and UTI, in the negative binomial regression model, there were more genes being downregulated than being upregulated. These down-regulated genes were enriched in the neutrophil involved immunity such as neutrophil activation and degranulation, indicating the immunomodulatory effects of UTI is mediated *via* regulation of neutrophil activity. Neutrophil is a kind of innate immune cell, acting as forerunners to clear the infection and resolute the inflammation during sepsis. Neutrophil extracellular traps (NETs) have been demonstrated to kill the pathogens by releasing DNA decorated with histone and granular proteins. However, unregulated NETs have a significant influence on the pathogenesis of sepsis-induced multiple organ damage, including arterial hypotension (shock), hypoxemia, renal, neurological, coagulopathy, and hepatic dysfunction (51). In our study cohort, patients in the UTI group showed more severe circulatory shock and organ dysfunction on day 1 (e.g. higher serum lactate and SOFA), but the duration of vasopressor dependent days were similar between the two groups. This results indicate beneficial effects of UTI for ameliorating the severity of organ dysfunctions. Biological process of neutrophil degranulation is closely related to the NET formation and we deduce that the effects of UTI could be mediated *via* reducing the incidence of NET formation. To the best of our knowledge, there has been no study exploring the effects of UTI on NET formation, and can be explored in further experimental assays.

To further corroborate the results from the bulk RNA-seq data, single cell RNA-seq was performed to identify component cell types of PBMC. The cellular components of the bulk RNA-seq samples can be decomposed with the scRNA-seq matrix as reference. Interestingly, our study found that the MDSC, which shared morphological similarity to neutrophils, were significantly expanded with the UTI treatment. It is well known that MDSC had anti-inflammatory effects that can ameliorates organ injury (45). In our study, the enriched biological processes in MDSC comparing UTI versus control samples were those associated with suppressed inflammatory responses.

Finally, we explored cell-cell communication patterns across cell types in UTI and control samples. Some conventional signaling pathways associated with immunomodulatory potency, such as ANNEXIN, GRN and RESISTIN, were identified to be differentially regulated in UTI versus control groups in our study. For example, many studies have shown that GRN signaling plays an essential role in sepsis immunity, including bacterial clearance, cell growth and survival, tissue repair, and the regulation of inflammation (52–54). Our study showed that the reduced incoming GRN signaling in neutrophils is control specific, indicating that UTI is associated with restoration of GRN signaling.

Several limitations must be acknowledged in the study. Firstly, the study was observational in nature and the treated and control groups were not fully exchangeable. However, we tried to adjust for confounding factors between the two groups by multivariable regression model in the DESeq2 pipeline. The study can be considered as hypothesis-generating and the findings need to be validated in experimental studies. Second, the subjects were enrolled from multiple centers in the study and the batch effects can confounding potential biological effects. We used DESeq2 pipeline to address this issue.

## Data Availability Statement

The datasets presented in this study can be found in online repositories. The names of the repository/repositories and accession number(s) can be found below: https://ngdc.cncb.ac.cn/omix/release/OMIX005600 (publicly accessible); and https://ngdc.cncb.ac.cn/bioproject/browse/PRJCA006118 (under controlled access because of ethical and privacy restrictions. Requests to access the datasets should be directed to the corresponding author/s).

## Ethics Statement

The study was approved by the ethics committee of Sir Run Run Shaw hospital (approval number: 20201014-39). The patients/participants provided their written informed consent to participate in this study.

## Author Contributions

YH, ZZ, and GZ designed research studies, SJ, LC, MY, CG, YiY, GD, and WZ conducted experiments, JZ, GH, LQ, JW, YX, JS, NW, MW, YaY, YiY, YT, JH, QB, and LX acquired data, HC and ZZ analyzed data, and ZZ and YH wrote the manuscript. All authors contributed to the article and approved the submitted version.

## Funding

The study was supported by Health Science and Technology Plan of Zhejiang Province (2021KY745), National Natural Science Foundation of China (81901929) and Key Research & Development project of Zhejiang Province (2021C03071). LC received funding from Zhejiang Provincial Project of Medical and Health Technology (2022PY099), Youth Foundation of Jinhua Municipal Central Hospital (JY2020-2-10) and Key Project of Jinhua City (2021-3-037) and Key Laboratory of Emergency and Trauma (Hainan Medical University), Ministry of Education (Grant No. KLET-202118). Ulinastatin was provided by the Techpool Bio-Pharma Co., Ltd.

## Conflict of Interest

The authors declare that the research was conducted in the absence of any commercial or financial relationships that could be construed as a potential conflict of interest.

## Publisher’s Note

All claims expressed in this article are solely those of the authors and do not necessarily represent those of their affiliated organizations, or those of the publisher, the editors and the reviewers. Any product that may be evaluated in this article, or claim that may be made by its manufacturer, is not guaranteed or endorsed by the publisher.

## Correction note

A correction has been made to this article. Details can be found at: 10.3389/fimmu.2026.1793340.
