# Correction: Integrated single cell and bulk RNA-Seq analysis revealed immunomodulatory effects of Ulinastatin in sepsis: a multicenter cohort study

**DOI:** 10.3389/fimmu.2026.1793340

**Published:** 2026-02-03

**Authors:** Lin Chen, Senjun Jin, Min Yang, Chunmei Gui, Yingpu Yuan, Guangtao Dong, Weizhong Zeng, Jing Zeng, Guoxin Hu, Lujun Qiao, Jinhua Wang, Yonglin Xi, Jian Sun, Nan Wang, Minmin Wang, Lifeng Xing, Yi Yang, Yan Teng, Junxia Hou, Qiaojie Bi, Huabo Cai, Gensheng Zhang, Yucai Hong, Zhongheng Zhang

**Affiliations:** 1 Department of Critical Care Medicine, Affiliated Jinhua Hospital, Zhejiang University School of Medicine, Jinhua, China; 2 Department of Emergency, Zhejiang Provincial People’s Hospital, People’s Hospital of Hangzhou Medical College, Hangzhou, China; 3 The 2nd Department of Intensive Care Unit, The Second Affiliated Hospital of Anhui Medical University, Hefei, China; 4 Department of Critical Care Medicine, The First People’s Hospital of Changde City, Changde, China; 5 Department of Emergency Medicine, The First Affiliated Hospital of Harbin Medical University, Harbin, China; 6 Department of Critical Care Medicine, Zhuzhou Central Hospital, Zhuzhou, China; 7 Emergency Department, Shengli Oilfield Central Hospital, Dongying, China; 8 Department of Critical Care Medicine, The Second Affiliated Hospital of Xi’an Medical University, Xi’an, China; 9 Department of Critical Care Medicine, Lishui Center Hospital, Lishui, China; 10 Department of Critical Care Medicine, The Fourth Affiliated Hospital of Anhui Medical University, Anhui Medical University, Hefei, China; 11 Department of Emergency Medicine, Sir Run Run Shaw Hospital, Zhejiang University School of Medicine, Hangzhou, China; 12 Department of Emergency Medicine, The Second Hospital of Jiaxing, Jiaxing, China; 13 Department of Critical Care Medicine, The First Affiliated Hospital of Xi’an Jiaotong University, Xi’an, China; 14 Department of Emergency, Qingdao Municipal Hospital, QingDao University School of Medicine, Qingdao, China; 15 Department of Critical Care Medicine, Second Affiliated Hospital, Zhejiang University School of Medicine, Hangzhou, China; 16 Key Laboratory of Precision Medicine in Diagnosis and Monitoring Research of Zhejiang Province, Department of Emergency Medicine, Sir Run Run Shaw Hospital, Zhejiang University School of Medicine, Hangzhou, China

**Keywords:** sepsis, single cell, ulinastatin, RNA-seq, immunosuppression

The **Data Availability Statement** was erroneously given as “The datasets presented in this study can be found in online repositories. The name of the repository and accession number can be found below: China National Genomics Data Center; PRJCA006118 (https://ngdc.cncb.ac.cn/bioproject/browse/PRJCA006118)”. The correct **Data Availability Statement** is “The datasets presented in this study can be found in online repositories. The names of the repository/repositories and accession number(s) can be found below:

https://ngdc.cncb.ac.cn/omix/release/OMIX005600 (publicly accessible); and https://ngdc.cncb.ac.cn/bioproject/browse/PRJCA006118 (under controlled access because of ethical and privacy restrictions. Requests to access the datasets should be directed to the corresponding author/s).”

The original version of this article has been updated.

