# Supplementary material for: Integrated Single Cell and Bulk RNA-Seq Analysis Revealed Immunomodulatory Effects of Ulinastatin in Sepsis: A Multicenter Cohort Study
Source: Front Immunol. 2022 May 11;13:882774. doi: 10.3389/fimmu.2022.882774 (PMC9130465; doi:10.3389/fimmu.2022.882774)
Supplement: Supplementary file 1 [file DataSheet_1.pdf]

Supplemental digital content for

Integrated single cell and bulk RNA-seq analysis revealed immunosuppressive effects of ulinastatin in sepsis: a multicenter cohort study

Table of Contents

Methodology ***2***

Detailed methods for WGCNA **2**

Enrichment analysis for biological function and transcription factors **2**

Identification of miRNA-target interactions **3**

Results ***4***

Weighted gene co-expression network analysis (WGCNA) **4**

eFig 1. Sample clustering and clinical trait heatmap 4

eFig 2. Summary network indices (y-axes) as functions of the soft thresholding power (x-axes) 5

eFig 3. Histogram of the Area under the cumulative recovery curve. 6

Single cell RNA-seq analysis **7**

eFig 4. Quality control of cells included for downstream analysis. 7

eFig 5. Clustering of single cell with different resolution. 8

eFig 6. UMAP visualization of individual cells stratified by sample identity 9

eFig 7. Bar plot showing enriched gene ontology terms for each type of cells. 10

Methodology

Detailed methods for WGCNA

The first step in constructing a weighted gene co-expression netwoek (WGCNA) was to choose the soft thresholding power to which co-expression similarity was raised to calculate adjacency. We chose from a set of values from 1 to 30 based on the criterion of approximate scale-free topology. Finally, modules were identified in the resulting dendrogram using the Dynamic Tree Cut algorithm. This algorithm has several advantages such as capability of identifying nested clusters and flexibility. Modules with similar expression profiles were merged at the threshold of 0.25. Gene significance was defined as the student t-test statistic for testing differential expression between ulinastatin (UTI) and controls. The significance level was adjusted for multiple testing with Bonferroni correction.

Module eigengene was calculated for each module as the first principal component of gene expressions for that module. Correlation analysis was performed to relate module eigengene to external traits including age, sex, SOFA, days and UTI group.

Gene significance for UTI group was correlated to the module membership to investigate whether genes significantly associated with UTI group was also associated with module membership. Module membership (eigengene-based connectivity) for each gene was calculated by correlating its gene expression profile with the module eigengene of a given module. For a given module, a module membership value of 0 indicates that a gene is not part of the module, whereas a module membership of − 1 or 1 is highly connected to the module.

Enrichment analysis for biological function and transcription factors

Modules associated with important clinical trait such as UTI group were further analyzed for their enrichment in Gene Ontology (GO) pathways. Specifically, the gene set from a given module were enriched to GO terms to find whether some of functional GO terms are over-represented using annotations for that gene set. Dot plot shows the gene ratio and adjusted p values for each enriched GO terms. Enriched terms were organized into a network with edges connecting overlapping gene sets. In this way, mutually overlapping gene sets are tend to cluster together, making it easy to identify functional modules. The category net plot depicts the linkages of genes and GO terms as a network, which is helpful to see which genes are involved in enriched pathways and genes that may belong to multiple annotation categories.

Modules (gene lists) significantly correlated with the mortality trait were tested for its over-representation in transcription factor (TF) binding motifs by using RcisTarget. Two types of databases (i.e. Gene-motif rankings and the annotation of motifs to transcription factors) were employed in the analysis: Gene-motif rankings which provides the rankings of all the genes for each motif and the annotation of motifs to transcription factors. Parameter settings for the score of each pair of gene-motif were: species = Homo sapiens, Scoring/search space = 500 bp uptream the transcription start site (TSS), Number of orthologous species = 10. The annotation of motifs to transcription factors was performed using the motifAnnotations_hgnc ('mc9nr', 24,453 motifs).

Identification of miRNA-target interactions

The multiMiR package was employed for the retrieval of miRNA-target interactions from 14 external databases in R. These databases are comprehensive collections of predicted and validated miRNA-target interactions and their associations with diseases and drugs. The module of interest (related to UTI group) was those associated with mortality outcome. It was interesting to check whether some, or all, of these genes within a module were targeted by the same miRNA(s). We restricted our search to the “mirtarbase” table because this table included only experimentally validated miRNA-target interactions.

Results

Weighted gene co-expression network analysis (WGCNA)

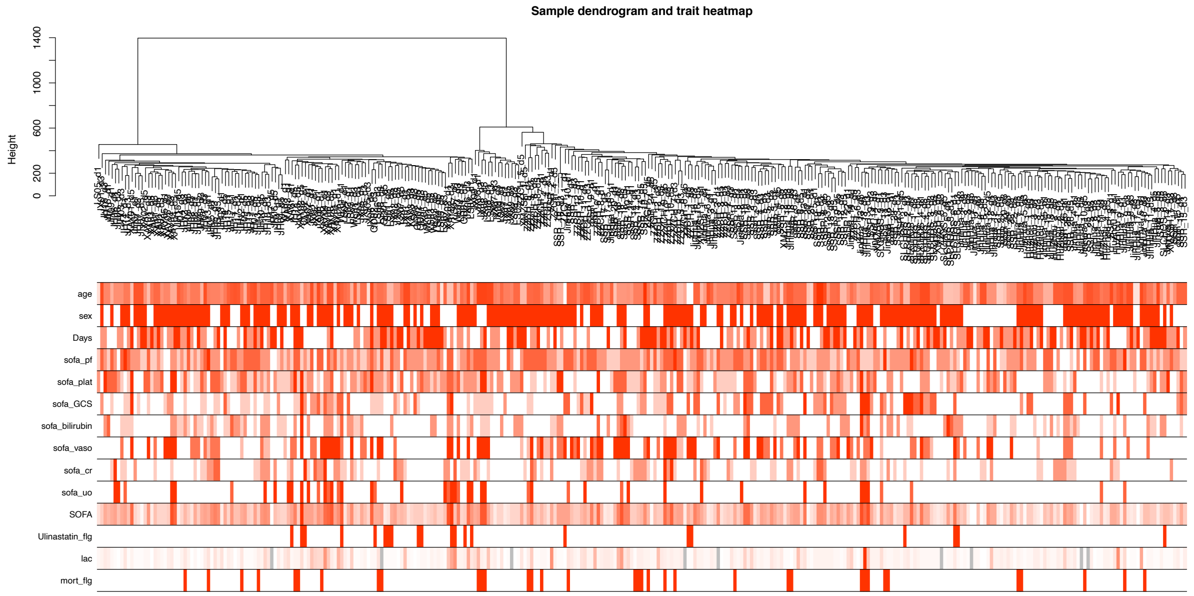

eFig 1. Sample clustering and clinical trait heatmap

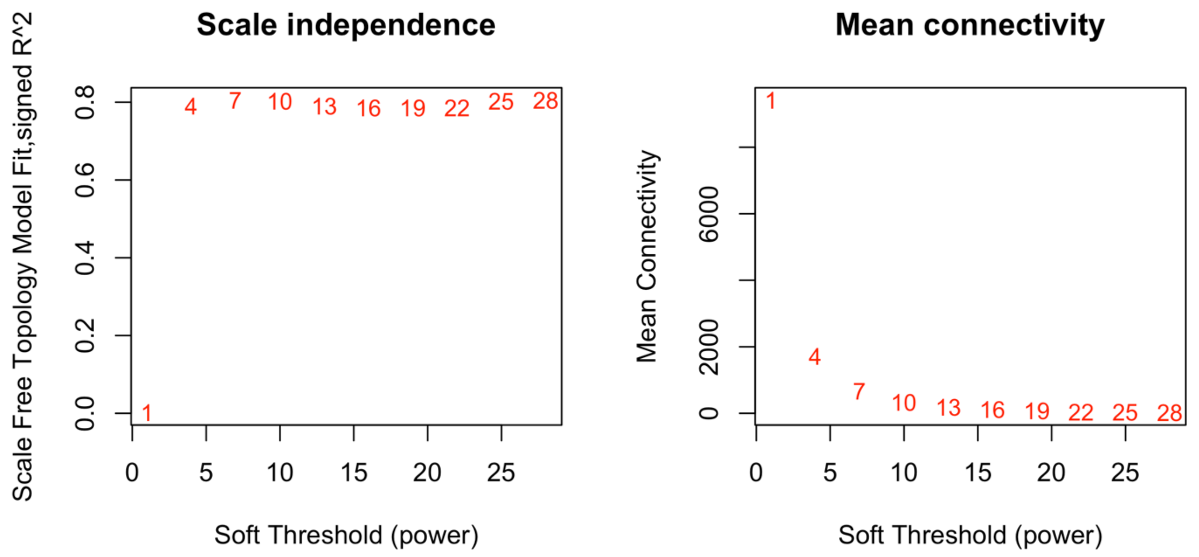

eFig 2. Summary network indices (y-axes) as functions of the soft thresholding power (x-axes). Numbers in the plots indicate the corresponding soft thresholding powers. The plots indicate that approximate scale-free topology is attained around the soft-thresholding power of 100 for the entire sepsis cohort. Because the mean connectivity measures decline steeply with increasing soft-thresholding power, it is advantageous to choose the lowest power that satisfies the approximate scale-free topology criterion (R2 > 0.8).

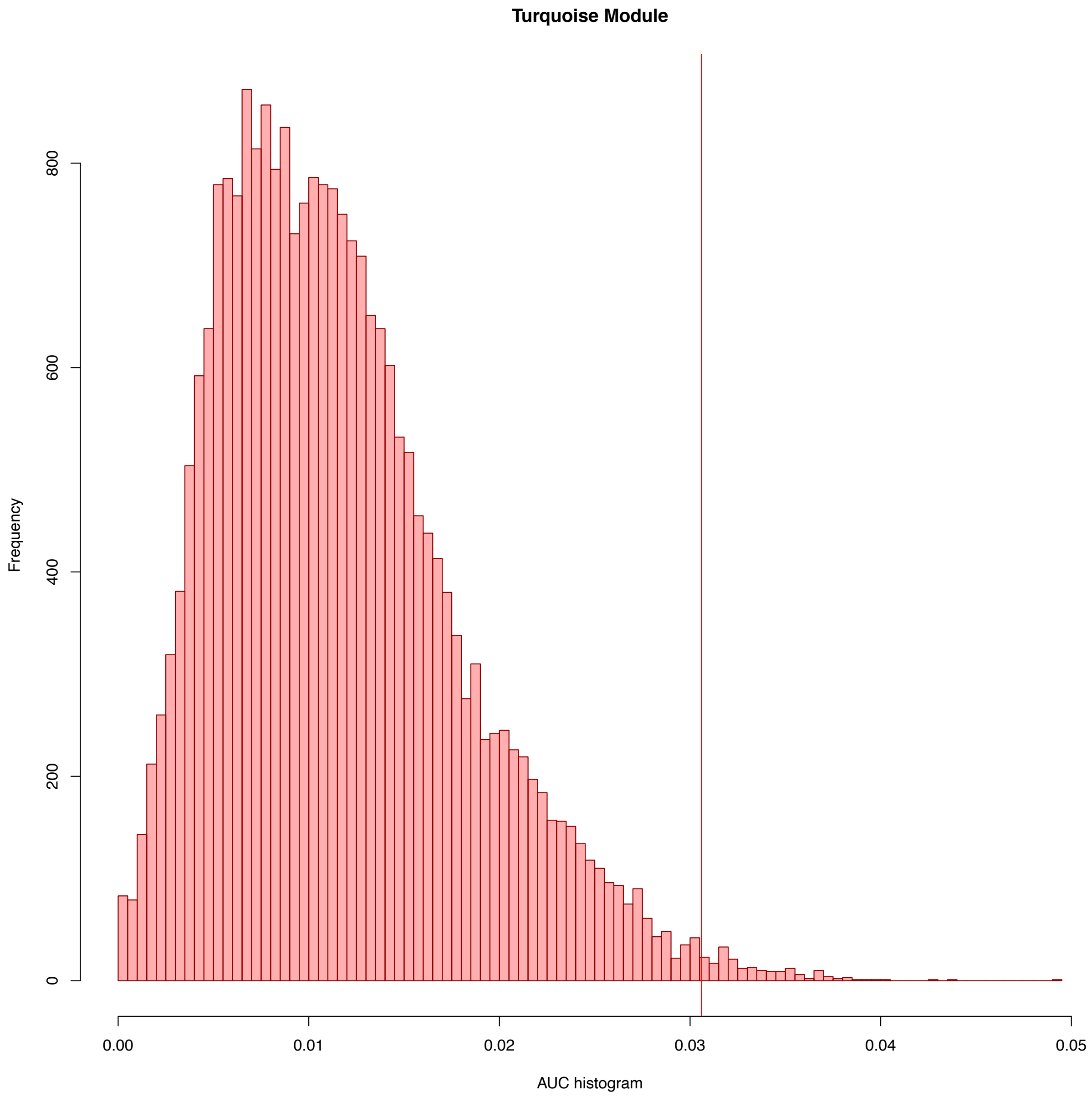

eFig 3. Histogram of the Area under the cumulative recovery curve.

The first step to estimate the over-representation of each motif on the black module gene-set is to calculate the Area Under the Curve (AUC) for each pair of motif-gene set. This is calculated based on the recovery curve of the gene-set on the motif ranking (genes ranked decreasingly by the score of motif in its proximity, as provided in the motifRanking database). The red vertical line indicates the significance level that motifs with an AUC greater than the significance level are considered significant motifs.

Single cell RNA-seq analysis

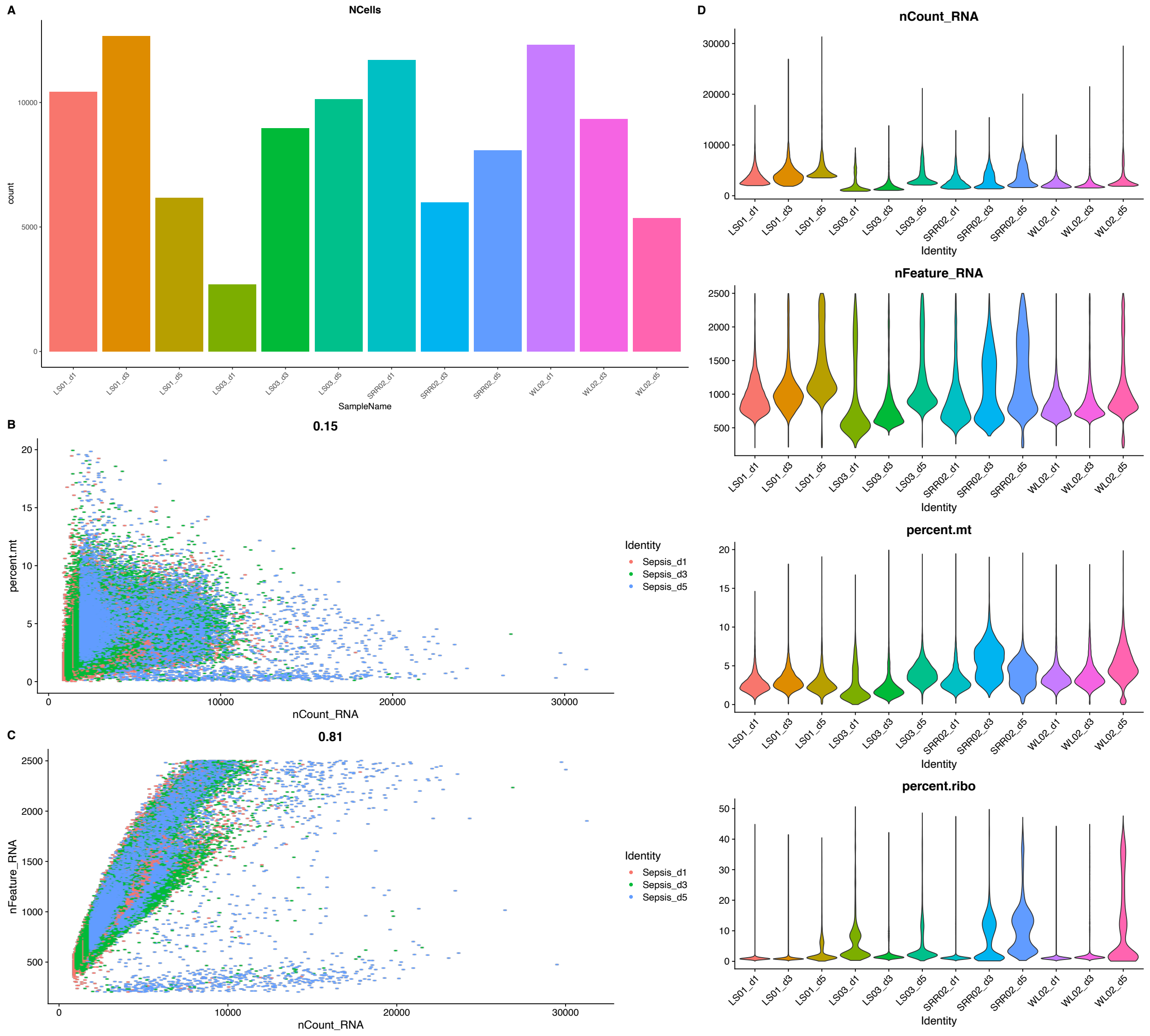

eFig 4. Quality control of cells included for downstream analysis.

A) Distribution of the number of single cells in all samples. B) percentage of mitochondria genes versus number of RNA counts, points were colored by the days. C) scatter plot of number of RNA features against RNA counts. D) violin plots showing the distribution of some important quantities such as number of RNA feature, count, mitochondria RNA percentage and ribosome RNA percentage.

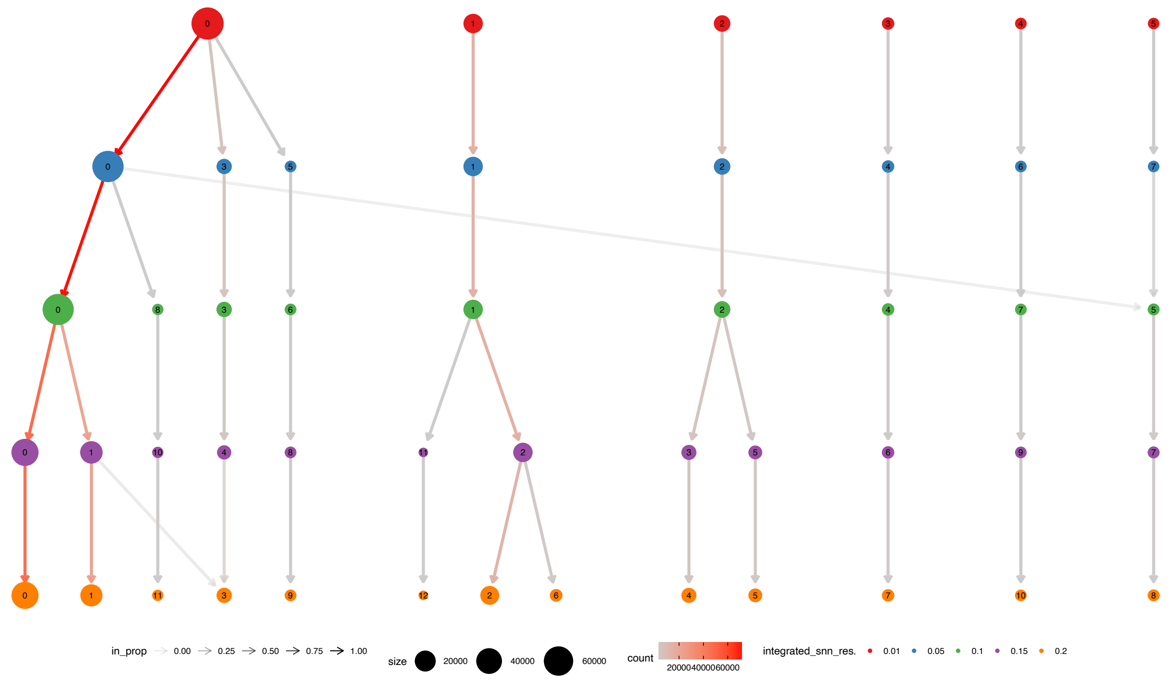

eFig 5. Clustering of single cell with different resolution.

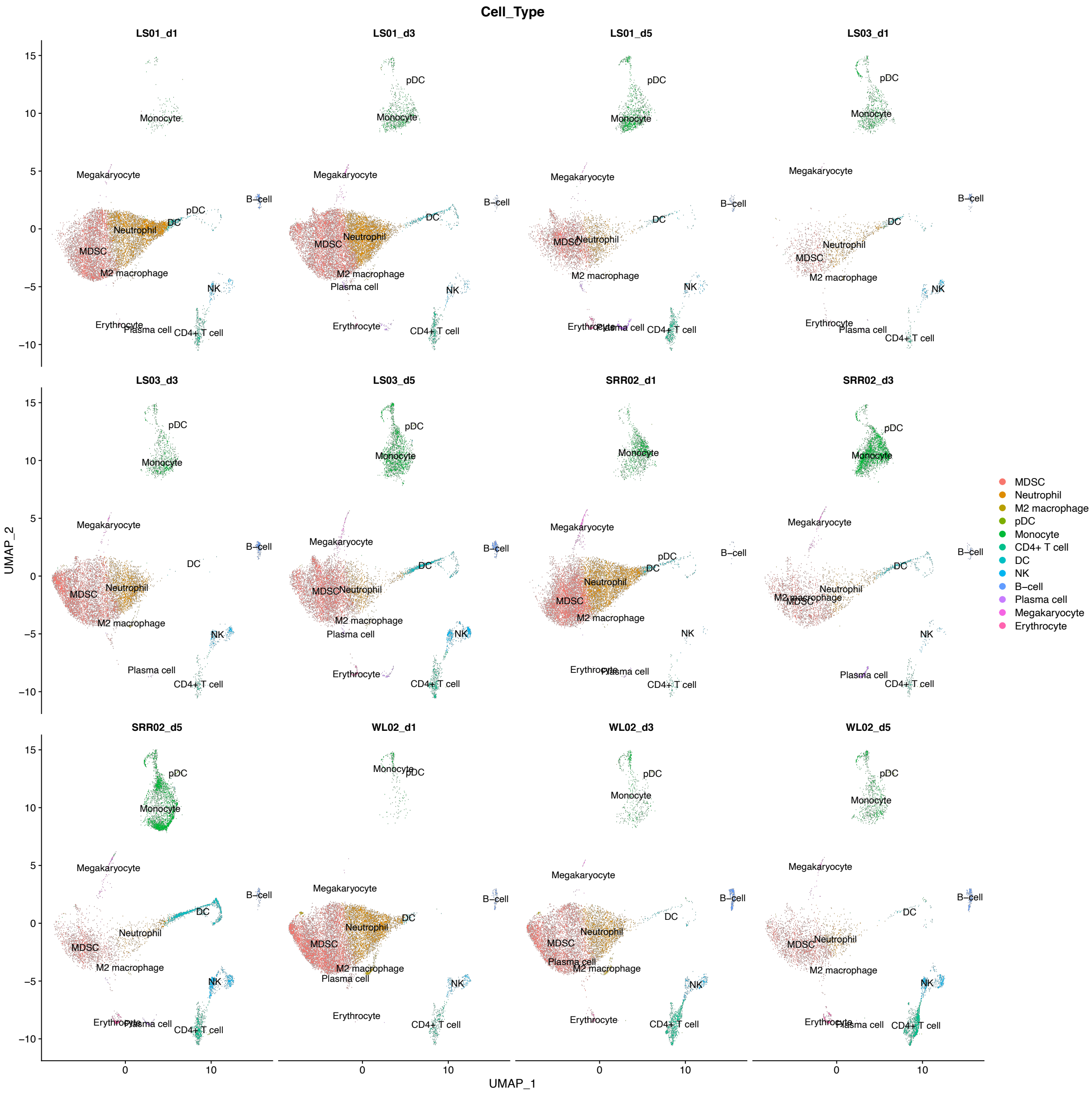

eFig 6. UMAP visualization of individual cells stratified by sample identity.

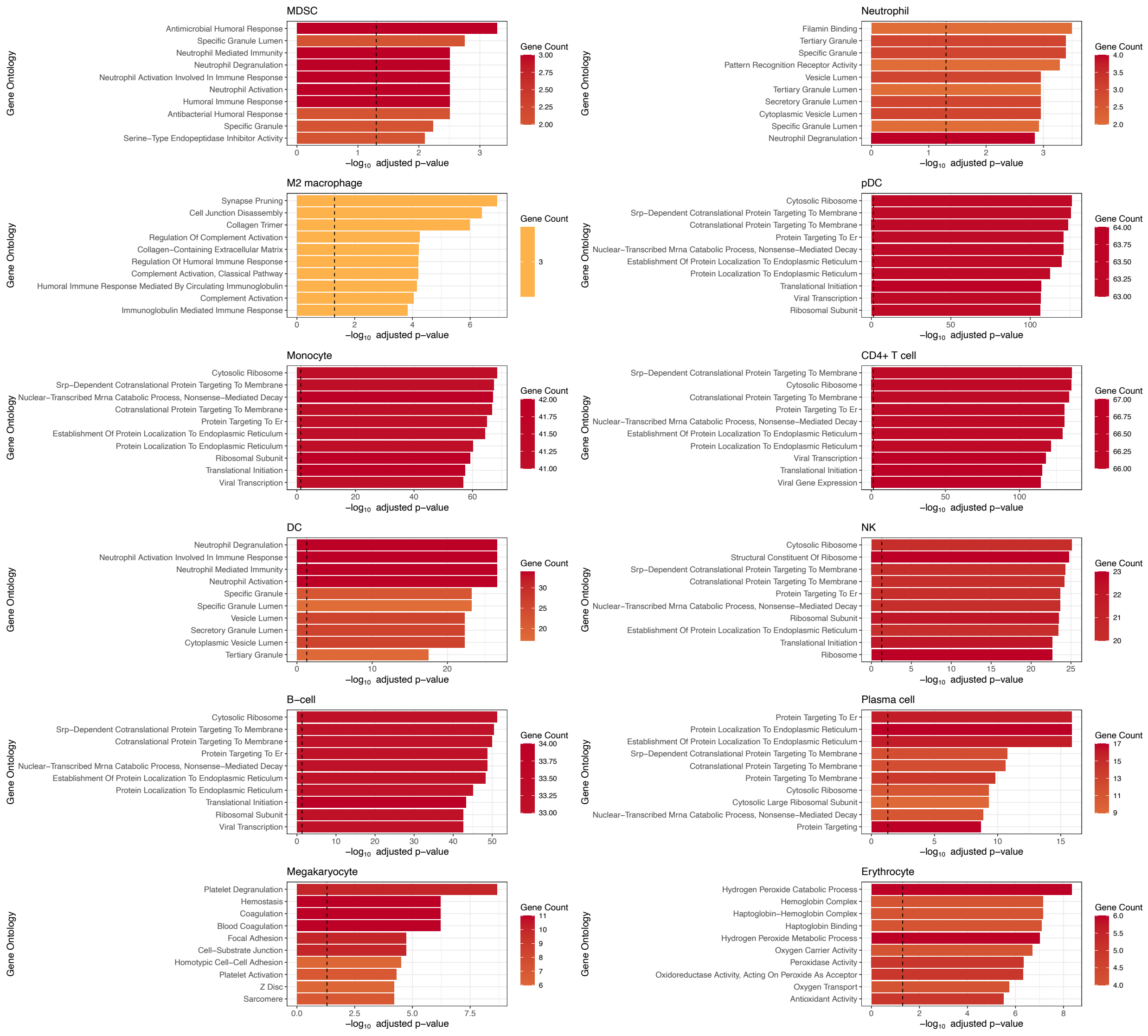

eFig 7. Bar plot showing enriched gene ontology terms for each type of cells.
